# Cryo-EM structure of a protein nanopore reveals lipid acyl chains as integral components

**DOI:** 10.1126/sciadv.aeg9162

**Published:** 2026-10-30

**Authors:** Milton Ashworth, Oliver W. Noble, Michael J. Plevin, Oliver W. Bayfield, Alfred A. Antson

**Affiliations:** ^1^ York Structural Biology Laboratory, Department of Chemistry, University of York, York YO10 5DD, UK.; ^2^ York Biomedical Research Institute, University of York, York YO10 5DD, UK.; ^3^ Department of Biology, University of York, York YO10 5DD, UK.

## Abstract

Nanopore-based technologies are revolutionizing nucleic acid sequencing and molecular sensing, yet the structural basis by which pore-forming proteins interact with lipid membranes remains poorly defined. The mycobacterial porin MspA is a benchmark biological nanopore owing to its exceptional stability and uniquely narrow constriction, but accurate structural information is limited to protein-only models. Here, we present a 2.5-angstrom resolution cryo–electron microscopy structure of the M2 mutant of MspA embedded in a phospholipid nanodisc, providing a high-resolution experimental observation of MspA in a lipid environment. The structure reveals an annular arrangement of extended lipid acyl chains surrounding the MspA oligomer and contacting the transmembrane barrel. Their resolved densities indicate a substantially more ordered annular lipid environment than would be expected from a largely heterogeneous and nonspecific protein-lipid interface. The structure also demonstrates how specific M2 MspA mutations modulate pore polarity, influencing ion selectivity and transport. By determining the membrane-embedded structure of MspA and revealing an ordered arrangement of acyl chains forming extensive hydrophobic interactions with the protein, this study demonstrates the importance of the surrounding lipid environment in the design of protein nanopores.

## INTRODUCTION

Nanopore sensing is used in a wide array of fields, including health care, ecology, and biosciences, where it plays an essential role in advancing both fundamental research and biotechnological applications (*1*–*3*). Nanopore nucleic acid sequencing has introduced substantial improvements over earlier methods, enabling ultralong read lengths and direct detection of DNA/RNA modifications (*4*–*6*). Moreover, the potential applications of nanopores extend beyond nucleic acid sequencing. Studies using the *Mycobacterium smegmatis* porin A (MspA) have demonstrated its versatility in peptide detection (*7*), characterization of posttranslational modifications (*8*), sugar sensing (*9*), identification of rare earth elements (*10*, *11*), analysis of nucleoside analogs (*12*), aptamer interaction studies (*13*), discrimination of enantiomers (*14*), and detection of DNA-peptide conjugates (*15*, *16*).

MspA is widely used in nanopore design owing to its exceptional stability and, critically, its short and narrow constriction, which is essential for accurate nanopore sensing (*17*, *18*). MspA is a homo-octameric protein, containing a wide hydrophobic β barrel that readily inserts into lipid bilayers. In its natural role, this MspA barrel functions as a channel facilitating the diffusion of small hydrophilic molecules across the outer mycobacterial membrane (*19*). However, despite its role in solute translocation across a membrane, initial sequencing trials with wild-type MspA were hindered by frequent pore blockages. To enhance DNA capture and translocation efficiency, several engineered versions were designed, most notably the M2 MspA mutant (*20*). This protein variant contains four point mutations within the central tunnel (D90N, D91N, D93N, and D118R) and two at the outer rim (D134R and E139K) that improve pore performance. Structural data to date have been limited to protein variants lacking these six M2 mutations. Furthermore, the previously determined wild-type structure [Protein Data Bank (PDB): 1UUN] differs from the M2 variant by an additional A96R mutation introduced to facilitate crystallization (*21*).

The rationale for mutations in the central tunnel was to alter the electrostatic potential to enhance DNA translocation efficiency (*20*). The M2 MspA variant has been extensively characterized using biochemical approaches and shown to be applicable for achieving single-nucleotide resolution during DNA sequencing, including detection of noncanonical bases (*20*, *22*–*24*). More recent advances include the development of single polypeptide chain M2 MspA protein constructs, enabling asymmetric configurations and even alteration in subunit number (*25*). However, experimental structural characterization of these engineered protein variants has not been carried out. Moreover, no experimental structure of any MspA protein variant has been determined in a lipid bilayer environment. This leaves a critical gap in fully understanding protein-lipid interactions, the influence of lipid environment on protein conformation, and the structural effects of the six introduced mutations. In the absence of experimental data, molecular dynamics (MD) simulations were widely used to inform pore reengineering (*26*, *27*). In the case of MspA, MD studies have highlighted that displacement of water molecules, particularly at the constriction site, is a key contributor to the ionic current that underpins single molecule resolution (*28*). While MD simulations are powerful predictive tools, the conclusions derived are most valuable when analyzed in combination with detailed structural information obtained from experimental data, which are important for validating the accuracy of MD models, predictions of pore behavior, and more effective reengineering strategies.

Here, we report a 2.5-Å cryo–electron microscopy (cryo-EM) structure of M2 MspA protein embedded in a nanodisc, composed of 1-palmitoyl-2-oleoyl-sn-glycero-3-phosphocholine (POPC), a zwitterionic phospholipid that provides a native-like bilayer environment. The atomic model reveals key residues involved in lipid interactions and provides high-resolution structural details of the internal tunnel and pore constriction, including protein-solvent interactions. Notably, MspA induces organization within the surrounding lipid environment, with four lipid acyl chains per subunit (32 per protein oligomer) occupying defined positions around the transmembrane barrel and forming extensive hydrophobic interactions with the protein. Furthermore, we show that the recombinant MspA M2 protein, produced in *Escherichia coli*, contains a posttranslational modification at pore-lining His148 (N^1^ methylation). This modification, previously unreported, has potential implications for nanopore function, given the widespread use of this construct in nanopore applications. By resolving the structure of M2 MspA in a lipid bilayer context, this study provides a structural foundation for understanding nanopore function and for informed engineering strategies that explicitly consider protein-lipid interactions.

## RESULTS

### Overall structure

Cryo–electron micrographs and two-dimensional (2D) class averages (Fig. 1, A and B) demonstrated embedding of MspA oligomers into nanodiscs. Single-particle reconstruction imposing C_8_ symmetry resulted in maps (Fig. 1, C and D) with 2.5-Å resolution (Table 1), revealing the structure of the octameric oligomer and enabling building an atomic model for the M2 MspA protein along with an adjacent layer of lipid molecules interacting with the protein.

**Fig. 1.**
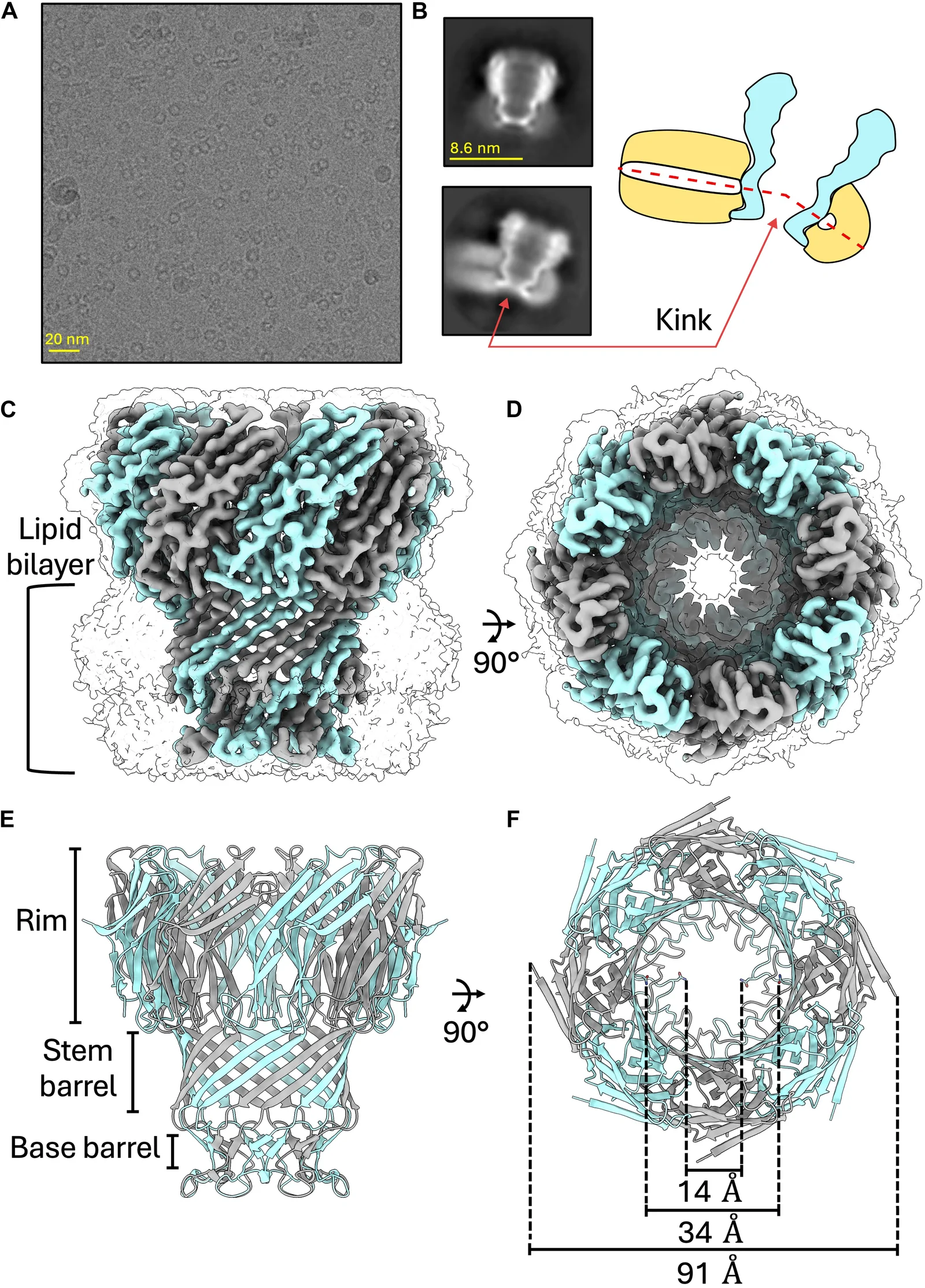
Structure of M2-MspA embedded in a lipid nanodisc. (**A**) Representative cryo-EM micrograph. (**B**) 2D class averages of MspA nanodisc particles along with a cartoon illustrating how MspA induces a kink, visible in the red dashed line drawn along the lipid bilayers (yellow) on both sides of the protein molecule (cyan). (**C** and **D**) Cryo-EM density maps viewed from the side (C) and along the central axis (D). Density corresponding to the lipidic bilayer [labeled in (C)] surrounds the stem barrel and the base barrel. (**E**) Ribbon diagram with the alternating MspA chains colored differently and with the base/stem barrels and rim subdomains labeled. (**F**) Ribbon diagram viewed along the central axis, showing diameters of key oligomeric features. Side chains extending into the central tunnel and defining its internal diameters of 14 Å (Asn^91^) and 34 Å (Asn^79^) are highlighted.

**Table 1.** Cryo-EM data collection and refinement statistics.

Data collection	
Voltage/kV	200
Magnification	240,000
Defocus range/μm	1.6–0.6
No. micrographs	3565
Pixel size/Å	0.574
Dose rate/pixel s^−1^	50
Exposure time	2.57
No. EER fractions	12
Data processing and refinement	
Symmetry	*C*_8_
Pixel size/Å	0.8
No. particles	82082
Map resolution (FSC = 0.143)/Å	2.5
Map sharpening B-factor/Å^2^	−56.0
Map-model correlation	0.87
Protein residues (per chain)	184
Ligands (acyl molecules)	32
Water molecules (per chain)	100
R.M.S.D. bond length/Å	0.003
R.M.S.D. bond angles/°	0.5
Ramachandran plot	
Favored/%	97.8
Allowed/%	2.2
Outlier/%	0.0

The MspA oligomer is composed of three regions: the rim, the stem barrel, and the base barrel. The MspA rim domain (residues 1 to 71 and 123 to 184) sits atop two consecutive antiparallel β strands (72 to 83 and 112 to 121) which make up the stem barrel. The stem barrel strands are joined by segments making up the base barrel (residues 83 to 111) (Fig. 1E).

The rim has an external diameter of 92 Å (as measured between Gly^1^ Cα atoms of opposite chains; Fig. 1F) and an internal diameter of 45 Å as measured between Arg^134^ sides chain moieties projecting toward the tunnel’s entrance at the top of the rim domain, where their positions are stabilized by ionic interactions of guanidinium moieties with Glu^59^ side chains (Fig. 2). The stem barrel consists of 16 β strands, with each monomer contributing two strands of 9 and 11 residues each, respectively. These strands run antiparallel, forming a wide internal barrel composed of 16 β strands. The narrowest region of the stem barrel tunnel, measuring 34 Å in diameter, is defined by side chains of Asn^79^ and Arg^118^ (Figs. 1F and 2), the latter being a mutation introduced in the M2 variant of the protein. The weaker and more diffused density of Arg^118^ side chains, compared to other amino acids inside the barrel, indicates their relative flexibility and ability to attain alternative conformations. The base barrel of MspA includes the constriction point, where the internal diameter of 14 Å is defined by a ring of Asn^91^ side chains, stabilized by hydrogen bonding interactions with the adjacent ring of Asn^90^ residues (Fig. 2). In the wild-type protein, both 90 and 91 positions are occupied by negatively charged aspartic acids. Cryo-EM maps revealed an additional density adjacent to the imidazole group of His^148^ (Fig. 2). Accordingly, mass spectrometry identified N^1^ methylation of His^148^ (fig. S1), which accounts for the observed density.

**Fig. 2.**
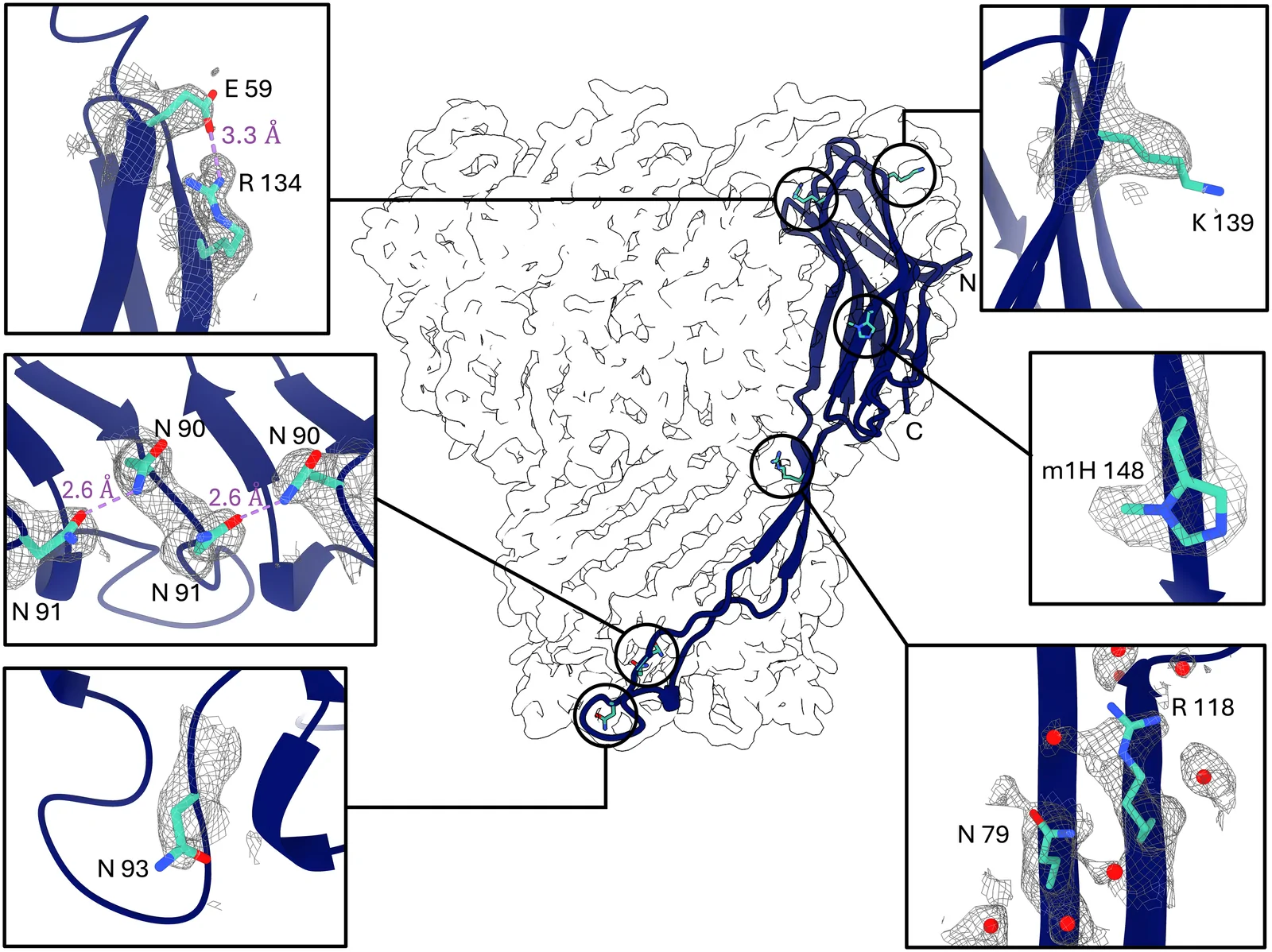
Molecular details of M2-specific key differences to wild-type MspA. Cryo-EM density (center) is shown as transparent molecular surface overlaid with ribbon diagram of a single subunit (N and C refer to N and C termini) and showing side chains of M2-specific amino acids, which are separately shown in expanded images. Expanded images for Arg^134^ and Arg^118^ also depict adjacent charged/polar residues affected by the introduced mutations. Solvent molecules are shown as red spheres.

### Protein-lipid interactions

Our choice of POPC phospholipids was dictated by this lipid yielding nanodiscs with a relatively narrow and reproducible size distribution, as well as its similar size and chemistry to the DPhPC (1,2-diphytanoyl-sn-glycero-3-phosphocholine) lipids most often used in nanopore experiments (*20*). The exterior surface of the stem and base barrels are hydrophobic, surrounded by density corresponding to lipids present within the nanodisc (Fig. 1). The density indicates that the upper leaflet of the lipid bilayer exclusively surrounds the stem barrel, while the lower leaflet occupies the surface around the base barrel (Fig. 3A). The lipid bilayer spans the entire length of the base and stem barrels, with the tunnel pore opening aligning flush with the membrane surface, not extending beyond the lipid layer.

**Fig. 3.**
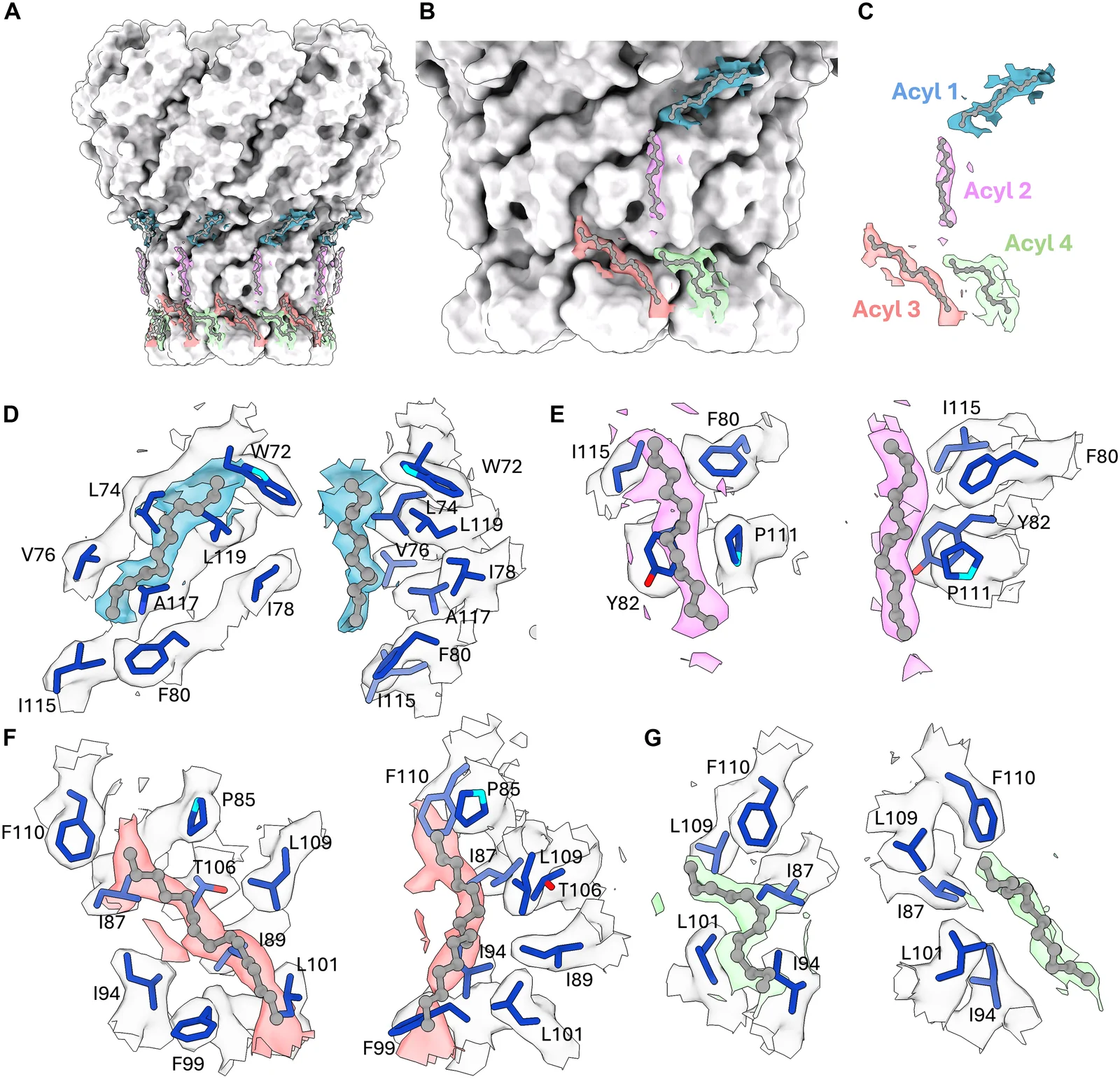
MspA-lipid interactions. (**A**) M2 MspA shown as molecular surface (white) with surrounding elongated cryo-EM densities, fitted with modeled segments of lipid acyl chains in (B) to (G). Lipid densities are colored blue (Acyl 1), magenta (Acyl 2), pink (Acyl 3), and green (Acyl 4). (**B**) Enlarged view at (A), showing only the four acyl chains for clarity, with acyl chains shown separately, with labels, in (**C**). (**D** to **G**) Cryo-EM densities corresponding to acyl chain ligands and interacting amino acid side chains, shown with overlaid atomic models, in two views related by ∼40° rotation around the vertical axis. Side-chain carbon atoms are in blue, oxygens in red, and nitrogens in cyan. Carbon atoms of acyl chains are in gray. Protein side-chain density is in white, with acyl chain models shown with corresponding densities colored as in (A) to (C), with 2.5-Å density cutoff around acyl chain models. For clarity, the colored lipid density is generated using a difference map (subtracting protein density).

Cryo-EM maps reveal several elongated densities lined next to exposed hydrophobic residues of the protein, which are well accounted for by four modeled lipid acyl chains, Acyl 1 to Acyl 4 on Fig. 3. There is no clear density corresponding to polar head groups of lipids, indicating their higher flexibility and potential conformational variability.

At the top layer of the membrane, a 12-carbon acyl chain (Acyl 1) makes hydrophobic interactions with eight amino acids (Trp^72^, Leu^74^, Val^76^, Ile^78^, Phe^80^, Ile^115^, Ala^117^, and Leu^119^; Fig. 3D). Acyl 2, containing 12 carbon atoms is nestled in a shallow groove formed between two adjacent protein chains, with side chains of Ile^115^ from one protein subunit and Phe^80^, Pro^111^, and Tyr^82^ from the neighboring subunit contributing to the binding site.

The exterior surface of the base barrel features a hydrophobic grove around its outside, which accommodates acyl chains of lipids, forming extensive hydrophobic interactions and defining their conformation (Fig. 3, A and B). The hydrophobic grove accommodates acyl chains comprising 15 carbon atoms (Acyl 3), which makes hydrophobic interactions with side chains of Pro^85^, Ile^87^, Ile^89^, Ile^94^, Phe^99^, Leu^101^, Thr^106^, Leu^109^, and Phe^110^ (Fig. 3F). Last, 12–carbon atom Acyl 4 (Fig. 3G) makes hydrophobic contacts with side chains of Ile^87^, Ile^94^, Leu^101^, Leu^109^, and Phe^110^.

### Comparison of M2 MspA with the wild-type protein

The overall structures of the M2 MspA embedded in the lipid bilayer resembles the structure of the wild-type protein determined by x-ray crystallography, with the Cα root mean square deviation (RMSD) of 0.38 Å and all-atom RMSD of 0.88 Å. The 71 to 121 residue segment forming the stem and base barrels showed even closer similarity, with the Cα RMSD of 0.25 Å and all-atom RMSD of 0.77 Å. These comparisons indicate that while membrane insertion preserves the overall conformation, it introduces differences in the side-chain conformations of amino acids.

M2 MspA exhibits substantial changes in pore polarity compared to the wild-type protein (Fig. 4A). In the wild type, the pore constriction is defined by the side chains of Asp^90^, Asp^91^, and Asp^93^, whereas in the M2 variant, these residues are replaced by asparagine, generating a markedly more neutral surface charge within the constriction. The atomic model of M2-MspA enables detailed analysis of the structure of this protein, in particular residues lining the pore and defining the constriction (Fig. 2). The side chains of mutated residues D90N/D91N/D93N align closely with their positions in the crystal structure (Fig. 2). The density corresponding to the side chain of Arg^118^ extends into the central tunnel (Fig. 2), in common with the wild-type Asp^118^ side chain, but its positively charged side chain adopts a different conformation, reoriented upward toward the rim domain. This amino acid substitution also affects Asn^79^, whose side chain adopts a different conformation in M2. The side-chain positions of additionally substituted D134R and E139K are consistent with their positions in the crystal structure, with Arg^134^ introducing a salt bridge interaction with the neighboring Glu^59^ (Fig. 2). Overall, while these mutations substantially alter electrostatic potential inside the pore, they do not affect the overall fold and shape of the protein.

**Fig. 4.**
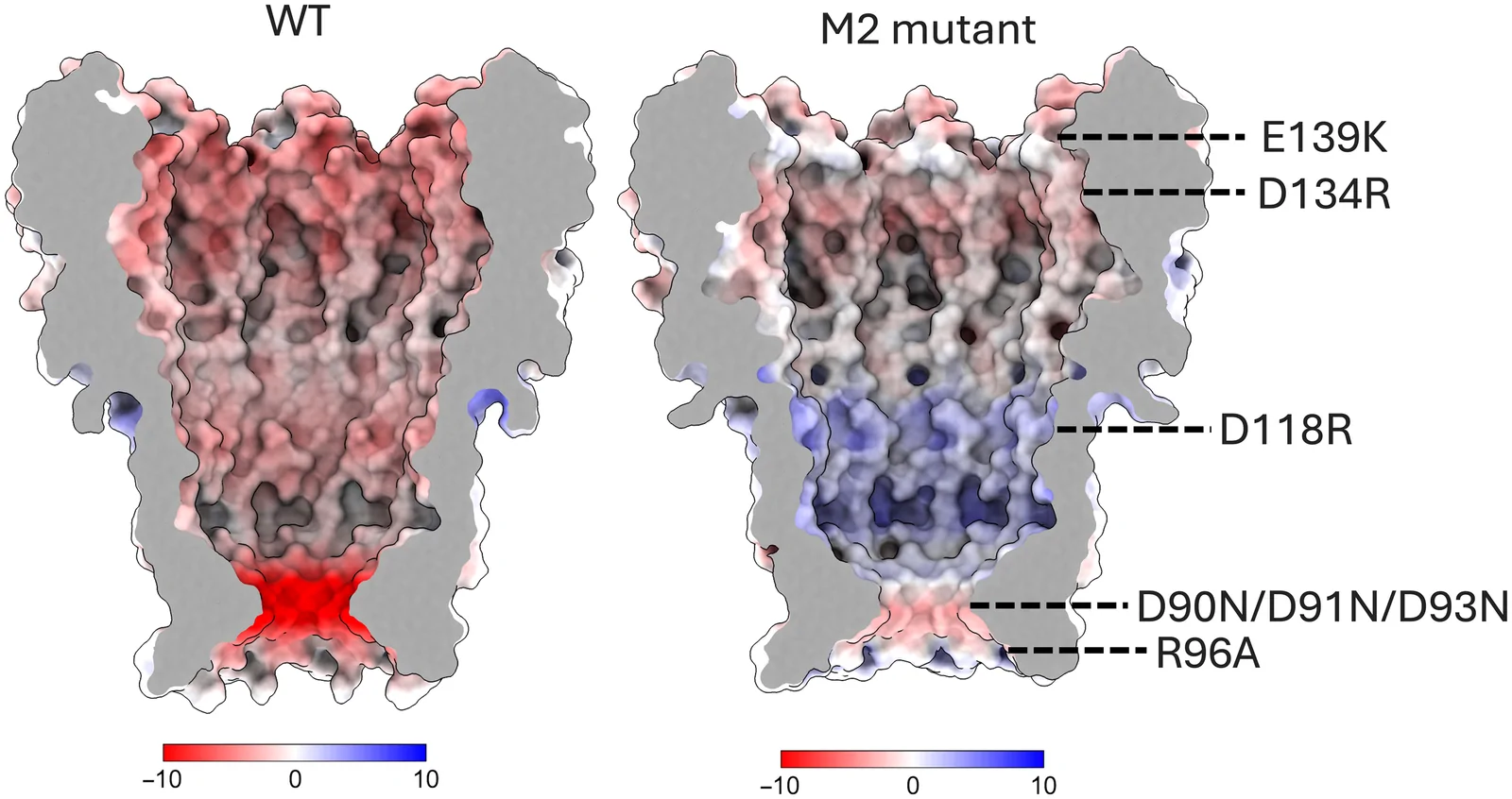
Comparison of the surface electrostatic potential of wild-type and M2 MspA. Cross-sectional views of the protein oligomers are shown as molecular surfaces [left: wild-type (WT) MspA, PDB: 1UUN; right: M2 MspA]. The surfaces are colored according to electrostatic potential and viewed from the center of the oligomer toward the internal tunnel surface. Red and blue correspond to negatively and positively charge regions, respectively.

## DISCUSSION

This study presents the experimentally determined structure of the M2 mutant of MspA pore (*25*, *29*) and the effects of mutations on the protein electrostatics and describes the structure of this protein within a membrane environment. The experimental density shows that the MspA presence leads to an ordered shell of lipid acyl chains, resulting in a defined protein-ligand assembly at the membrane interface. In particular, the structure uncovers hydrophobic interactions mediated by key surface residues of MspA that facilitate its membrane insertion, mediated by 19 amino acids per subunit (152 protein residues per oligomer) (Fig. 3). These hydrophobic interactions impose partial conformation order on the first lipid shell directly coordinated by the protein, distinguishing it from the far less ordered surrounding bilayer. The base β barrel containing the internal constriction forms a characteristic external groove that accommodates acyl chains Acyl 3 and Acyl 4 (Fig. 3, F and G), thereby defining their conformation. This groove likely acts as a structural template facilitating lipid positioning within the first annular shell. As the base β barrel also shapes the internal constriction of the central tunnel, protein-lipid interactions at its exterior surface, while stabilizing the overall structure, are expected to influence ionic current during DNA sequencing, particularly by affecting the baseline noise. Consistent with this, previous studies have reported considerable variability in current conductivity through MspA, likely arising from flexibility within the constriction region (*25*). While POPC lipids have not been used in most nanopore measurements conducted for MspA (*20*), POPC molecules have been previously used in MD simulations, to investigate protein-lipid interactions in a nanopore setting (*27*, *28*, *30*).

Notably, the substitution of Pro^97^, located at the base of the external surface with phenylalanine (P97F mutation) (*25*, *31*) previously shown to narrow channel conductance distribution, could stabilize protein-lipid interactions by introducing additional contacts with lipids and the adjacent Phe^99^, which interacts with the Acyl 3 ligand (Fig. 3F). Consistent with this, deletions within the loop containing Pro^97^ increased conductance fluctuations through the pore (*28*). Recent data also highlighted the importance of the C-terminal residues of MspA in maintaining protein stability, with addition of a C-terminal HisTag reducing the denaturation temperature (*17*). In addition to stabilizing intersubunit interactions within the octameric β barrel, the C terminus (Asn^184^; Figs. 2 and 3E) has been proposed to play an important role in protein folding and membrane integration (*17*). The data presented here allow to rationalize these findings: The carboxyl group of the C-terminal Asn^184^, not modeled in the previously reported structure (*21*), forms stabilizing salt bridge interaction with the guanidinium group of Arg^38^, and both of these groups are positioned at the top surface of the nanodisc density where the lipid head groups are likely located (Fig. 1C), stabilizing interactions with the membrane.

Previous studies reported compression of protein central tunnels in response to a lipid nanodisc environment (*32*), and conductance assays probing M2 MspA insertion into various block copolymer membranes suggested that membrane-induced protein compression affects the current readout (*33*). In contrast, the cryo-EM structure of nanodisc-embedded M2 MspA, presented here, reveals no compression in comparison with the crystal structure (1UUN) (*21*). This is reflected in the very low Cα RMSD of 0.38 Å between the M2 MspA structure presented here and the previously determined crystal structure of the wild type (*21*) protein, consistent with the exceptional intrinsic stability of MspA (*18*). While our data show no evidence of β barrel compression, they unveil that MspA imposes structural order on the extended acyl chains of surrounding lipid molecules. The cryo-EM structure shows that lipid acyl chains conform closely to the surface of MspA and adopt a conformation atypical to the wider bilayer, particularly at the external grove of the base β barrel, where they adopt a ∼45° tilted orientation relative to the tunnel axis (Fig. 3B). This enforced geometry likely affects the local membrane architecture as it induces a kink in the nanodisc, seen in the 2D class averages (Fig. 1B).

Previous MD simulation studies have explored lipid interactions with the surface of MspA (*16*, *27*, *34*, *35*), but most corresponding atomic coordinates are not in public domain, limiting direct comparison with data reported here. This issue has been ascribed to the absence of a dedicated global data repository for MD simulations resulting in MD datasets scattered across general repositories (e.g., Zenodo), lacking standardization of MD data and their limited/fragmented accessibility (*36*, *37*). The only MspA-lipid coordinates that we were able to access (*34*, *35*) differ substantially in the positioning of the lipid bilayer relative to the protein, often exhibiting direct protein-lipid atomic clashes (fig. S2). The experimental data reported here (Fig. 3) resolve the acyl chains of the first lipid shell around the protein, showing that lipids conform more closely to the protein surface, effectively mirroring the protein’s surface topology and adopting conformation that markedly differs from those reported earlier (fig. S2). Although annular lipids have traditionally been described as dynamically exchanging boundary lipids (*38*), with exchange rates increasing with distance from the protein (*39*), several studies have demonstrated the presence of discrete, high-affinity lipid binding pockets at membrane-exposed protein surfaces, preferentially occupied by lipids for prolonged times (*40*). High-resolution structure of connexin, a transmembrane gap junction protein, has revealed tightly organized annular lipids forming several ordered layers around the protein (*41*). Likewise, the MspA nanopore protein presents multiple lipid acyl chain binding sites repeated around the circumference of the oligomer, instigating formation of a coordinated and ordered ligand belt around its surface. In the structure presented here, the acyl chains, which form hydrophobic interactions with the protein, are resolved, while the headgroups lack well-defined density. This indicates that the MspA nanopore–lipid interaction may be driven primarily by acyl chains rather than by coordination of charged headgroups. This is consistent with prior observations that MspA readily inserts into bilayers of lipids with different head groups. These observations support a model with an at least partially ordered annular lipid shell, which adopts structural organization imposed by the protein. This concept is consistent with experimental structures of other nanopore-lipid protein complexes (*42*, *43*), which show that lipids adapt to the protein surface by tilting acyl chains at the protein surface, to maximize favorable interactions, further highlighting the capacity of the protein architecture to influence lipid behavior. In particular, cryo-EM density of the aerolysin pore (PDB: 9FM6) (*42*) reveals lipid density, surrounding the transmembrane β barrel (fig. S3) comparable to that resolved for MspA in the present study. Together, these observations demonstrate that lipid organization at the nanopore surface is not stochastic, as implied earlier (*34*, *35*, *38*) but structurally programmed, reinforcing the concept that membrane proteins can impose defined architectural constraints on their surrounding lipid environment.

Lipids coordinated with the base barrel exhibit slightly better-defined density than those at the stem barrel (Fig. 3), likely reflecting interactions with the relatively more homogeneous inner leaflet of the mycobacterial outer membrane (*44*). In contrast, the chemically diverse outer leaflet likely contributes to lower lipid occupancy and greater conformational variability around the stem barrel. In support of this, cryo-EM density corresponding to Phe^80^ shows that its side-chain aromatic ring occupies two alternative conformations, interacting with the acyl chains of Acyl 1 and Acyl 2, in contrast to a single conformation of Phe^80^ observed in the crystal structure, determined in the absence of bound lipids. These observations imply that variance in channel conductance may arise, at least in part, from torsional variation of protein side chains, such as Phe^80^. Future development of block copolymers for MspA should account for structural differences between the top and bottom leaflets, including the apparent ∼45° tilting adopted by the acyl chains of lipids at the base barrel (Fig. 3B). The MspA protein itself could be further mutated to enhance interactions with lipid acyl chains, in particular by introducing mutations at residues Val^76^, Ile^78^, and Ile^115^ (Fig. 3, D and E) that engage in hydrophobic interactions with the lipids.

The data presented here offer important insights about structural details of polar side chains introduced in the M2 variant of MspA. Notably, the side chain of introduced R118 (Fig. 2, D118R mutation) adopts a conformation that flips the orientation of N79 side chain, compared to the wild-type protein (*19*). Side chains of mutated D90N, D91N, and D93N substitutions, while preserving the conformations observed in the wild-type protein, alter the negative electrostatic potential of the pore’s tunnel at its most constricted part (Fig. 4). These structural differences are critical for understanding how M2 MspA alters the electrostatic environment and solvent accessibility, especially in the context of DNA interactions, where R118 was proposed to form a pocket with shape complementarity for thymine (*26*). An unexpected difference with the wild-type protein (PDB: 1UUN) (*19*) is the observation of a methylated H148, which is exposed inside the tunnel, occupying a site at the interface between each pair of adjacent subunits. This posttranslational modification appears to be unique for the M2 MspA, as both the M2 MspA used in this study and the wild-type protein (*21*) were produced using BL21 (DE3) *E. coli* cells. This M2 MspA modification has implications for nanopore function, given the widespread use of this construct in nanopore applications (*45*). In particular, the methylation raises the p*K*_a_ (where *K*_a_ is the acid dissociation constant) of this histidine, affecting its protonation state and hydrophobicity (*46*) and thus altering capture and interaction with analytes such as DNA.

In the M2 mutant structure presented here, several water molecules are observed in positions consistent with the crystal structure, indicating that lipid insertion does not displace all solvent molecules. Notably, more than 400 additional water molecules per oligomer, not previously reported for the wild-type protein crystallized in the presence of polyethylene glycol (*19*), are also identified in the cryo-EM structure presented here. Together with the identification of a partially ordered first annular lipid shell, these observations establish a detailed, experimentally derived structure of the membrane-embedded MspA nanopore. These findings further our understanding of MspA function and serve as a foundation for rational nanopore engineering, which explicitly considers protein-lipid-solvent interactions.

## MATERIALS AND METHODS

### Expression and purification of M2 MspA

The M2 variant of MspA includes six amino acid substitutions (D90N, D91N, D93N, R96A, D118R, D134R, and E139K) compared to the wild-type protein (*24*). A codon-optimized DNA sequence encoding residues 1 to 184 of M2MspA, with an N-terminal His-tag, was synthesized and cloned into the pET28a(+) expression vector (Twist Bioscience). The plasmid was resuspended in H_2_O to a concentration of 30 ng/μl and transformed into *E. coli* BL21-Gold (DE3) cells. Cultures were grown in LB at 37°C to an optical density (OD_600_) of 0.8, followed by induction with 0.5 mM isopropyl-β-d-thiogalactopyranoside (IPTG). Protein expression was carried out overnight at 18°C. Cells were harvested by centrifugation at 5000*g* for 15 min at 4°C, and the resulting pellets were stored at −80°C until use. For lysis, cell pellets were resuspended on ice at 5 g/ml in lysis buffer containing 150 mM NaCl, 50 mM tris-HCl (pH 7.8), 30 mM imidazole, and 10 mM *n*-dodecyl-β-d-maltoside (DDM). The suspension was incubated on ice for 15 min with gentle mixing, before transferring into 2-ml microcentrifuge tubes and heat treating at 95°C for 15 min. Following heat treatment, samples were centrifuged at 16,000*g* for 1 min, and the supernatants were pooled. The clarified lysate was applied to a 5-ml HisTrap HP column (Cytiva) at a flow rate of 3 ml/min. The column was washed with 15 column volumes of wash buffer [150 mM NaCl, 50 mM tris-HCl (pH 7.8), 30 mM imidazole, and 1 mM DDM] and eluted over a linear gradient of 500 mM imidazole. Eluted fractions were analyzed by SDS–polyacrylamide gel electrophoresis (SDS-PAGE), and those containing the target protein were pooled. To remove the His-tag, thrombin protease (200 U) was added, and the solution was dialyzed overnight at 4°C into 150 mM NaCl and 50 mM tris-HCl (pH 7.8). The cleaved protein was reapplied to a HisTrap HP column, and the flow-through, containing tag-free M2MspA, was collected. The protein was then concentrated using a 30-kDa molecular weight cutoff centrifugal spin filter to 4.3 mg/ml. The purified M2MspA protein was used fresh for nanodisc assembly, performed on the same day.

### Production and purification of MSP2N2 nanodisc scaffold protein

The plasmid encoding the nanodisc scaffold protein MSP2N2 was obtained from Addgene (a gift from S. Sligar; Addgene plasmid #29520; http://n2t.net/addgene:29520; RRID: Addgene_29520) (*47*–*49*). The construct was transformed into *E. coli* BL21-Gold (DE3) cells for expression. Cultures were grown in LB at 37°C to an OD_600_ of 0.8, then induced with 1 mM IPTG, and incubated overnight at 18°C. Cells were harvested by centrifugation at 5000*g* for 20 min at 4°C, and the pellets were stored at −80°C until purification. Frozen cell pellets were resuspended in buffer A containing 1% (v/v) Triton X-100, 300 mM NaCl, 40 mM tris-HCl (pH 8.0), and 0.25 mM EDTA. Protease inhibitors were added to a final concentration of 1 mM AEBSF, 1 mM leupeptin, and 1 mM pepstatin. Cells were lysed by sonication on ice, and the lysate was clarified by centrifugation at 38,000*g* for 20 min at 4°C. The supernatant was applied to a 5-ml HisTrap HP column (Cytiva) at a flow rate of 3.5 ml/min. Purification was performed according to previously published protocols (*49*, *50*). The column was sequentially washed with 20 column volumes (CV) of buffer A, 20 CV of buffer B [10 mM deoxycholate, 300 mM NaCl, and 40 mM tris-HCl (pH 8.0)], and 20 CV of buffer with no detergent [300 mM NaCl, 40 mM tris-HCl (pH 8.0), and 30 mM imidazole] before elution using 500 mM imidazole gradient. Elution fractions containing MSP2N2 were identified by SDS-PAGE, pooled, and diluted to a final NaCl concentration of 30 mM. The diluted protein was applied to a 1 ml QXL Sepharose ion exchange column (Cytiva) and eluted over a gradient of 300 mM NaCl. The protein was further purified by size exclusion chromatography using a Superose 6 Increase 10/300 column (Cytiva) equilibrated in 100 mM NaCl, 20 mM tris-HCl (pH 7.4), 2 mM EDTA, and 0.1 mM AEBSF. Fractions containing pure MSP2N2 were concentrated using a 10-kDa MWCO centrifugal concentrator to 15.0 mg/ml, flash-frozen in liquid nitrogen, and stored at −80°C until use.

### Assembly of M2 MspA in nanodiscs

Nanodisc assembly and reconstitution of MspA into MSP2N2 nanodiscs were performed with the POPC phospholipid, at the molar ratio of 1:3:300 of MspA octamer:MSP2N2: POPC. The threefold molar excess of the MSP2N2 nanodiscs was used to facilitate MspA insertion. The final concentrations of the nanodisc reaction mixture were as follows: MspA octamer, 12 μM; MSP2N2, 36 μM; POPC lipids, 10.8 mM; and deoxycholate, 21.7 mM.

Following detergent removal, the nanodisc mixture was applied to a Superose 6 Increase 10/300 column and equilibrated in 150 mM NaCl, 50 mM tris-HCl (pH 7.8) (fig. S4). Fractions from the assembled nanodisc peak were calculated to contain assembled nanodiscs at a concentration of 0.15 mg/ml based on ultraviolet absorbance and a combined extinction coefficient of 107720 M^−1^ cm^−1^.

### Cryo-EM grid preparation

Graphene oxide (GO) was deposited onto glow-discharged Quantifoil 300 mesh R1.2/1.3 grids (Quantifoil Micro Tools GmbH). Glow discharge was performed using a Pelco easiGlow device at 0.38 mBar pressure, 20 mA current, for 60 s with negative polarity. GO deposition was carried out as described earlier (*51*). Briefly, GO (2 mg/ml) was mixed in a 1:1 volume ratio with 1.5% (w/v) DDM. The mixture was vortexed and then diluted 1:200 in water, and 5 μl was applied to the surface of each grid. The back side of each grid was gently touched onto Whatman no. 1 filter paper to remove excess liquid. Prepared grids were used within 30 min.

Cryo-EM grid preparation was performed using a Vitrobot Mark IV (Thermo Fisher Scientific). A total of 3 μl of MspA M2 sample was applied to each GO-coated grid. After sample application, grids were incubated for 3 s before blotting. Blotting was carried out for 2 s using a blot force setting of −10. All blotting steps were conducted at a temperature of 4°C and humidity of 95%.

### Cryo-EM imaging

Data were collected at the University of York using the 200-kV Glacios instrument (Thermo Fisher Scientific), at a magnification of ×240,000, with movies recorded on a Falcon IV detector at 0.574 Å/pixel. Nominal defocus ranges for data collection were −1.8 to −0.4 μm. An objective aperture of 100 μm was used with a total accumulated dose of 50 e^−^/Å^2^. Micrographs were collected in EER format, with a total of 3565 images collected (Table 1).

### Single-particle 3D reconstruction and model refinement

Image data were processed using RELION 5.0 (fig. S5) (*52*, *53*). Motion correction of movie frames was performed using RELION’s built-in algorithm (*53*, *54*), with no grouping, binning, or frame patching applied. Contrast transfer function estimation was performed using CTFFIND4.1 (*55*), based on the power spectra generated from motion correction. Particle picking was performed using a TOPAZ model trained on a subset of 2D classes (*56*). Particles were extracted from dose-weighted micrographs at a box size of 512 pixels and then Fourier cropped to 352 pixels, corresponding to a final pixel size of 0.835 Å/pixel (1.45× scaling).

Following extraction, particles were subjected to 2D classification, and those corresponding to MspA nanodiscs were selected for further 3D classification. A 10-Å low-pass filtered map of PDB: 1UUN was used as the initial reference (*21*). A soft-edged mask of the protein was generated in RELION from the refined volume and used in subsequent refinements to calculate solvent-flattened Fourier shell correlation (FSC) curves. Postprocessing was used to generate B-factor sharpened maps by multiplying the soft mask with one of the half-maps from the refinement. During this step, the modulation transfer function of the detector and the calibrated pixel size were supplied. Cryo-EM density was further improved following nonuniform refinement in cryoSPARC (*57*). For the deposited protein-lipid structure, PDB entry 9S15, based on 82,082 particles, the final resolution was 2.5 Å, as defined by the FSC = 0.143 criterion (*58*).

Initial atomic models were generated using ModelAngelo (*59*). Model rebuilding was performed using Coot 0.9.8.95 (*60*), real-space refinement, and building of water molecules was performed using Phenix 1.21.2 (*61*). The data refinement statistics are shown in Table 1. All figures were generated using ChimeraX (*62*).

### LCMS

Excised protein bands were reduced and alkylated with 200 μl of 10 mM dithioerythrietol and 50 mM iodoacetamide, respectively. Protein content was digested using 100 ng of sequencing-grade trypsin (Promega, V5111). Peptides were loaded onto Evo Tip Pure tips for desalting and into disposable trap column for nanoUPLC using an EvoSep One system. A preset EvoSep 100 SPD gradient (from Evosep One HyStar Driver 2.3.57.0) was used with an 8-cm EvoSep C18 Performance column (8 cm by 150 μm by 1.5 μm). The nanoUPLC system was interfaced to a timsTOF HT mass spectrometer (Bruker) with a CaptiveSpray ionization source (Source). Positive PASEF-DDA, ESI-MS, and MS^2^ spectra were acquired using Compass HyStar software (version 6.2, Bruker). Instrument source settings were as follows: capillary voltage, 1700 V; dry gas, 3 liters/min; dry temperature, 180°C. Spectra were acquired between mass/charge ratio 100 and 1700. TIMS settings were as follows: 1/K0 0.6 to 1.60 Vs/cm^2^; ramp time, 100 ms; ramp rate, 9.42 Hz. Data-dependent acquisition was performed with 10 PASEF ramps and a total cycle time of 1.17 s. An intensity threshold of 2500 and a target intensity of 20,000 were set with active exclusion applied for 0.4 min postprecursor selection. Collision energy was interpolated between 20 eV at 0.6 V.s/cm^2^ and 59 eV at 1.6 Vs/cm^2^. Mascot generic files were generated by Compass Data Analysis version 6.1. Searches were carried out with Mascot version 3.1.0. Carbamidomethyl (C) was selected as a fixed modification. Variable modifications included acetyl (N-term), deamidated (NQ), Gln → pyro-Glu (N-term Q), Glu → pyro-Glu (N-term E), methyl (H), oxidation (M), and phospho (ST). A peptide mass tolerance of 15 parts per million combined with 0.05-Da tolerance for fragments was used for searches. The maximum number of missed cleavages considered was 2.

### Data deposition

The atomic model and corresponding cryo-EM map have been deposited with the PDB (accession code 9S15) and EMDB (accession code EMD-54442), respectively. Experimental cryo-EM data are available through EMPIAR (accession code EMPIAR-13060).
