# Supplementary material for: Cryo-EM structure of a protein nanopore reveals lipid acyl chains as integral components
Source: Sci Adv. 2026 Oct 30;12(44):eaeg9162. doi: 10.1126/sciadv.aeg9162 (PMC13721976; doi:10.1126/sciadv.aeg9162)
Supplement: Supplementary file 1 [file sciadv.aeg9162_sm.pdf]

Science Advances
AAAS

Supplementary Materials for

Cryo-EM structure of a protein nanopore reveals lipid acyl chains as integral components

Milton Ashworth *et al.*

Corresponding author: Oliver W. Bayfield, oliver.bayfield@york.ac.uk; Alfred A. Antson, fred.antson@york.ac.uk

Sci. Adv. **12**, eaeg9162 (2026)
DOI: 10.1126/sciadv.aeg9162

This PDF file includes:

Figs. S1 to S5

A

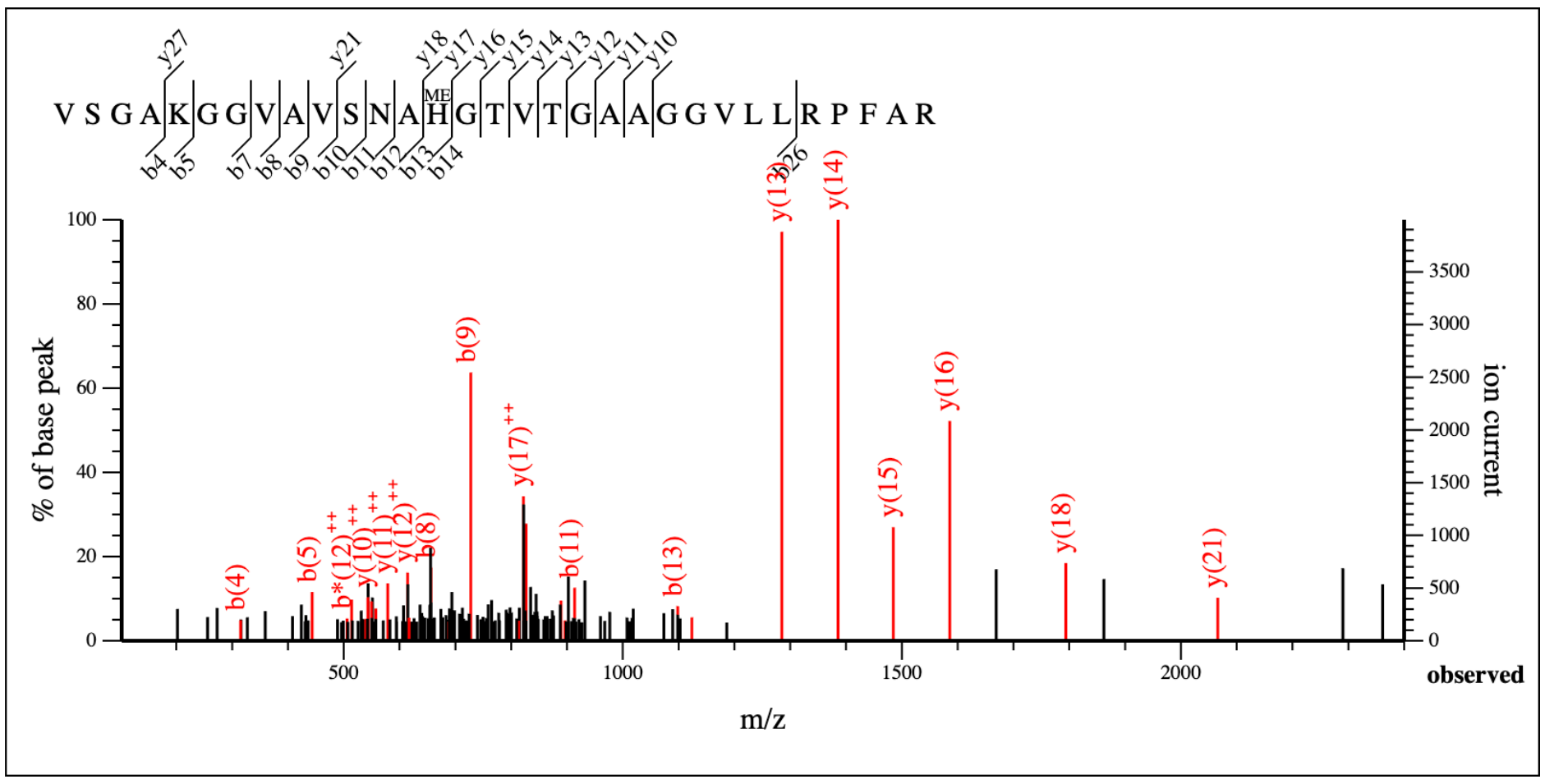

B

Monoisotopic mass of neutral peptide Mr(calc): 2890.5890
Fixed modifications: Carbamidomethyl (C) (apply to specified residues or termini only)
Variable modifications:
H14 : Methyl (H)
Ions Score: 23 **Expect:** 0.0067

https://biolmascot1.york.ac.uk/help/peptide_view_help.html

#	b	b^{++}	b*	b^{*++}	b^0	b^{0++}	Seq.	y	y^{++}	y*	y^{*++}	y^0	y^{0++}	#
1	100.08	50.542					V							31
2	187.11	94.058			169.1	85.052	S	2792.5	1396.8	2775.5	1388.3	2774.5	1387.8	30
3	244.13	122.57			226.12	113.56	G	2705.5	1353.3	2688.5	1344.7	2687.5	1344.2	29
4	315.17	158.09			297.16	149.08	A	2648.5	1324.7	2631.4	1316.2	2630.5	1315.7	28
5	***443.26***	222.13	426.23	213.62	425.25	213.13	K	2577.4	1289.2	2560.4	1280.7	2559.4	1280.2	27
6	500.28	250.65	483.26	242.13	482.27	241.64	G	2449.3	1225.2	2432.3	1216.7	2431.3	1216.2	26
7	557.3	279.16	540.28	270.64	539.29	270.15	G	2392.3	1196.7	2375.3	1188.2	2374.3	1187.7	25
8	***656.37***	328.69	639.35	320.18	638.36	319.68	V	2335.3	1168.2	2318.3	1159.6	2317.3	1159.1	24
9	***727.41***	364.21	710.38	355.7	709.4	355.2	A	2236.2	1118.6	2219.2	1110.1	2218.2	1109.6	23
10	***826.48***	413.74	809.45	405.23	808.47	404.74	V	2165.2	1083.1	2148.2	1074.6	2147.2	1074.1	22
11	***913.51***	457.26	896.48	448.75	895.5	448.25	S	***2066.1***	1033.6	2049.1	1025.1	2048.1	1024.6	21
12	1027.6	514.28	1010.5	505.77	1009.5	505.27	N	1979.1	990.05	1962.1	981.54	1961.1	981.05	20
13	***1098.6***	549.8	1081.6	541.29	1080.6	540.79	A	1865.1	933.03	1848	924.52	1847	924.02	19
14	1249.7	625.34	1232.6	616.82	1231.7	616.33	H	***1794***	897.51	1777	889	1776	888.51	18
15	1306.7	653.85	1289.7	645.33	1288.7	644.84	G	1642.9	***821.97***	1625.9	813.46	1624.9	812.97	17
16	1407.7	704.37	1390.7	695.86	1389.7	695.37	T	***1585.9***	793.46	1568.9	784.95	1567.9	784.46	16
17	1506.8	753.9	1489.8	745.39	1488.8	744.9	V	***1484.9***	742.94	1467.8	734.43	1466.9	733.93	15
18	1607.9	804.43	1590.8	795.92	1589.8	795.42	T	***1385.8***	693.4	1368.8	684.89	1367.8	684.4	14
19	1664.9	832.94	1647.8	824.43	1646.9	823.93	G	***1284.8***	642.88	1267.7	634.37			13
20	1735.9	868.46	1718.9	859.94	1717.9	859.45	A	1227.7	***614.37***	1210.7	605.86			12
21	1806.9	903.98	1789.9	895.46	1788.9	894.97	A	1156.7	***578.85***	1139.7	570.34			11
22	1864	932.49	1846.9	923.97	1846	923.48	G	1085.7	***543.33***	1068.6	534.82			10
23	1921	961	1904	952.48	1903	951.99	G	1028.6	514.82	1011.6	506.31			9
24	2020.1	1010.5	2003	1002	2002	1001.5	V	971.61	486.31	954.59	477.8			8
25	2133.1	1067.1	2116.1	1058.6	2115.1	1058.1	L	872.55	436.78	855.52	428.26			7
26	2246.2	**1123.6**	2229.2	1115.1	2228.2	1114.6	L	759.46	380.23	742.44	371.72			6
27	2402.3	1201.7	2385.3	1193.2	2384.3	1192.7	R	646.38	323.69	629.35	315.18			5
28	2499.4	1250.2	2482.4	1241.7	2481.4	1241.2	P	490.28	245.64	473.25	237.13			4
29	2646.4	1323.7	2629.4	1315.2	2628.4	1314.7	F	393.22	197.12	376.2	188.6			3
30	2717.5	1359.2	2700.5	1350.7	2699.5	1350.2	A	246.16	123.58	229.13	115.07			2
31							R	175.12	88.063	158.09	79.55			1

Supplementary Figure S1. Mass spectrometry identification of N¹-methylation at H148 residues. (A) Spectrum for the peptide fragment containing H148. (B) Raw ion table values for spectra.

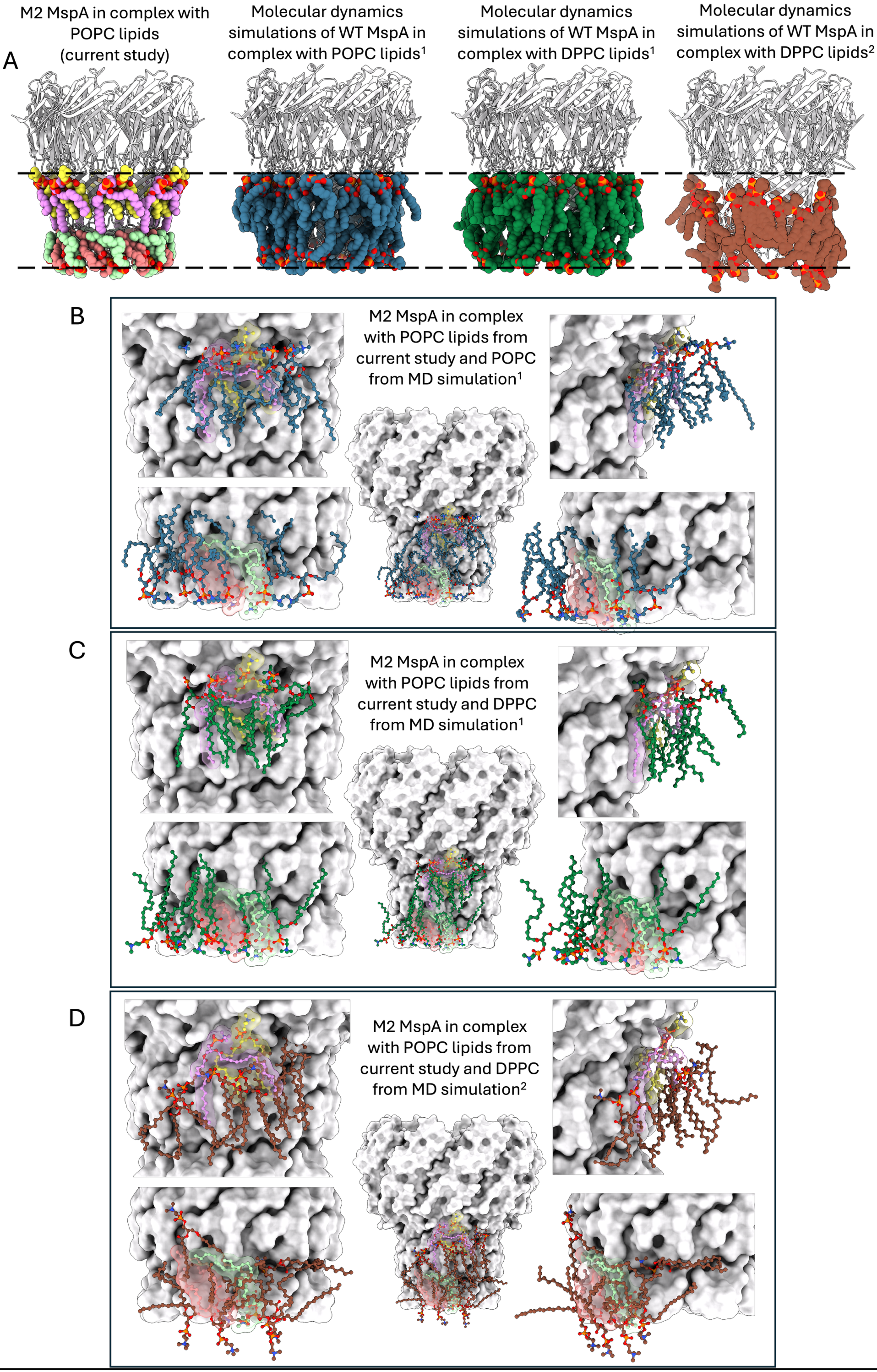

Supplementary Figure S2. Comparison of protein-lipid interactions with previously reported data derived by molecular dynamics simulations[1, 2]. (A) Position of the lipid bylayer with respect to the protein. Black dashed line indicates the upper and lower membrane leaflets as defined by experimental cryo-EM maps reported here, with protein shown as ribbon diagram (white), and boundary lipids (the closest to the protein surface) shown as van der Waals models coloured by atom type. POPC lipids derived from present cryo-EM study (left panel) are coloured as in Figure 3 of the main text. (B–D) Protein is shown as molecular surface (white). For clarity, only lipids (ball-and-stick) interacting with a single protein chain, correspond to a snapshot from each MD simulation, are shown. Lipids derived from the experimental structure of M2 MspA are overlayed and are coloured as in Figure 3

in the main text, shown along with corresponding molecular surface (semi-transparent). Carbon atoms of lipids derived from MD simulations are shown in different colours: (B) blue (POPC)[1], (C) green (DPPC)[1] and (D) terracotta (DPPC)[2].

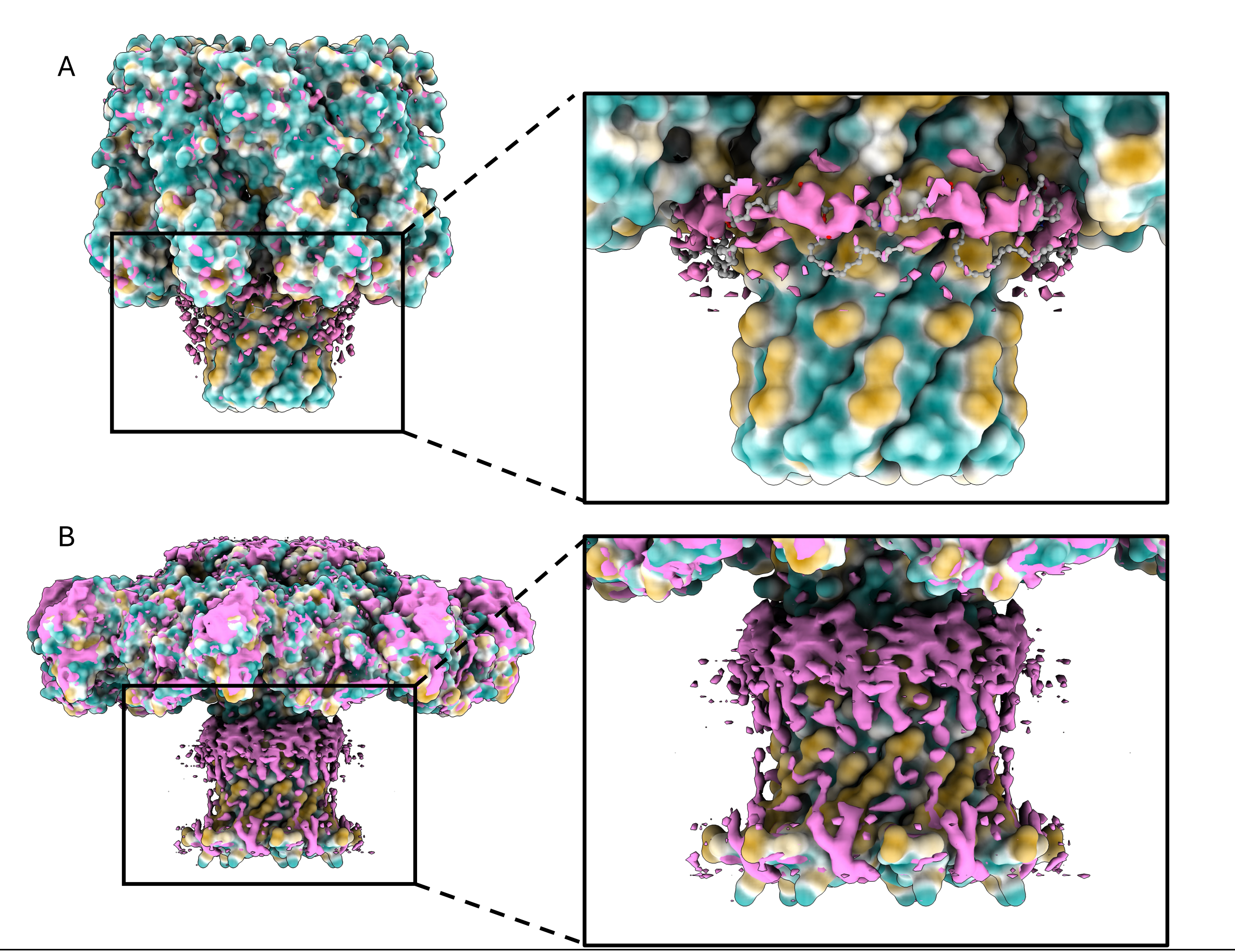

Supplementary Figure S3. Protein molecular surfaces coloured by hydrophobicity are shown for (A) α-Hemolysin (PDB: 9KRE) and (B) Aerolysin (PDB: 9FM6) [3, 4]. The corresponding cryo-EM density maps are overlaid in magenta, with a Gaussian filter (1 standard deviation) applied in ChimeraX. Expanded views of the lipid-associated density are shown for both proteins (pink); lipids modelled for α-Hemolysin are shown in panel A (grey sticks). Hydrophobicity changes from yellow (hydrophobic) to blue (hydrophilic).

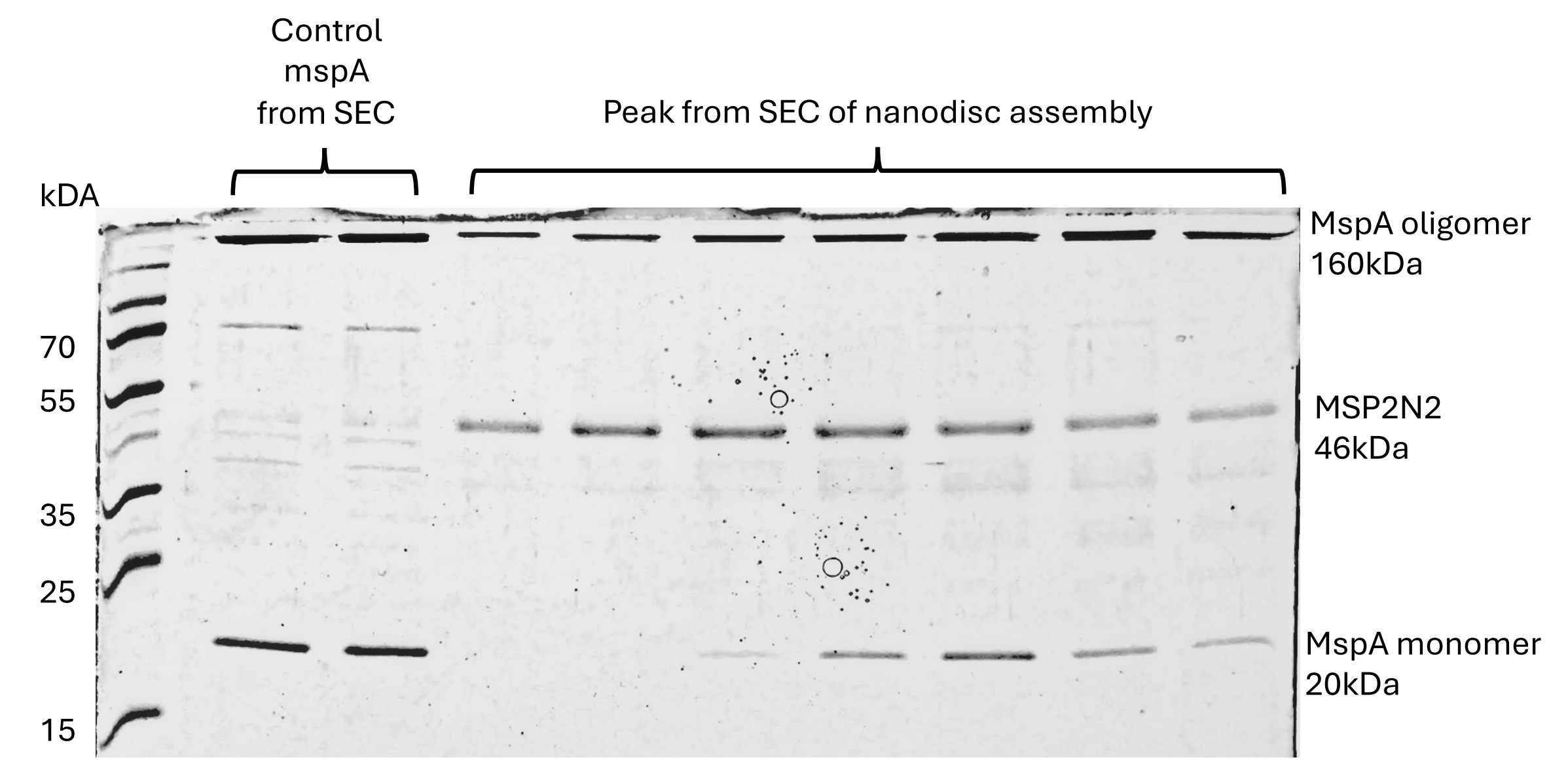

Supplementary Figure S4. SDS-PAGE of eluted fractions from “Superose 6 10/300 Increase” gel filtration column, demonstrating co-elution of MSP2N2 and MspA proteins.

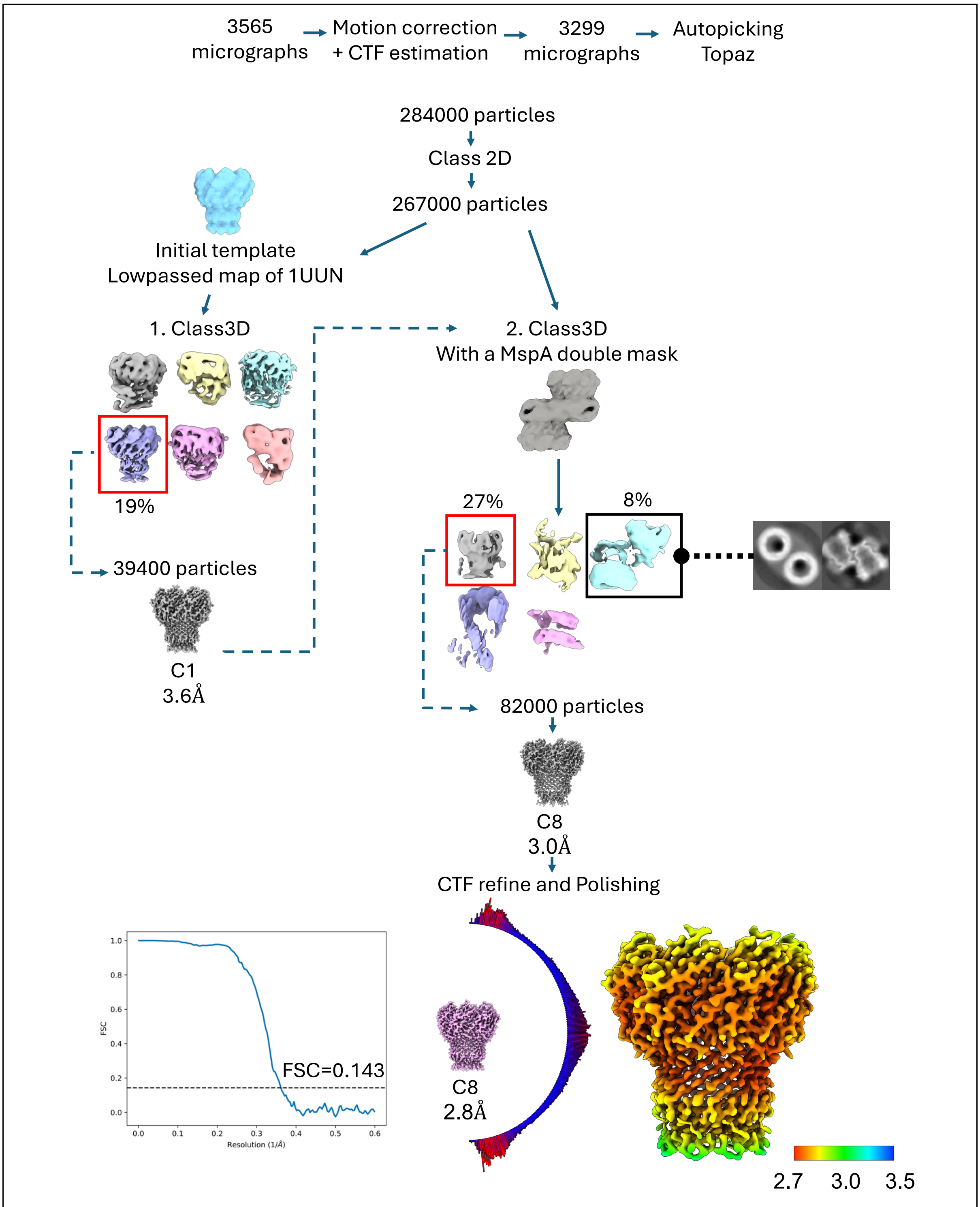

Supplementary Figure S5. Schematic representing the cryo-EM processing pipeline used for deriving 2.8Å resolution density map for MspA M2 embedded in nanodisc. The second 3D classification step (right), with a mask corresponding to two MspA oligomers inserted into the same nanodisc, was used for removing these particles from further processing. FSC curve with the FSC cut-off value of 0.143 was used to estimate resolution in the final reconstruction. Angular distribution of particles involved in the final reconstruction is shown with respect to the final map. The final map coloured by local resolution (red to blue) is also shown.
